# Acid–base disturbances in nephrotic syndrome: analysis using the CO_2_/HCO_3_ method (traditional Boston model) and the physicochemical method (Stewart model)

**DOI:** 10.1007/s10157-017-1387-8

**Published:** 2017-03-13

**Authors:** Tomomichi Kasagi, Hirokazu Imai, Naoto Miura, Keisuke Suzuki, Masabumi Yoshino, Hironobu Nobata, Takuhito Nagai, Shogo Banno

**Affiliations:** 1 Division of Nephrology and Rheumatology, Department of Internal Medicine, Aichi Medical University School of Medicine, Nagakute, Japan; 2 Aichi Medical University School of Medicine, Nagakute, Aichi 480-1195 Japan

**Keywords:** Acid–base disturbance, Metabolic acidosis, Nephrotic syndrome, Renin–angiotensin–aldosterone system, Respiratory alkalosis

## Abstract

**Background:**

The Stewart model for analyzing acid–base disturbances emphasizes serum albumin levels, which are ignored in the traditional Boston model. We compared data derived using the Stewart model to those using the Boston model in patients with nephrotic syndrome.

**Methods:**

Twenty-nine patients with nephrotic syndrome and six patients without urinary protein or acid–base disturbances provided blood and urine samples for analysis that included routine biochemical and arterial blood gas tests, plasma renin activity, and aldosterone. The total concentration of non-volatile weak acids (A_TOT_), apparent strong ion difference (SIDa), effective strong ion difference (SIDe), and strong ion gap (SIG) were calculated according to the formulas of Agrafiotis in the Stewart model.

**Results:**

According to the Boston model, 25 of 29 patients (90%) had alkalemia. Eighteen patients had respiratory alkalosis, 11 had metabolic alkalosis, and 4 had both conditions. Only three patients had hyperreninemic hyperaldosteronism. The Stewart model demonstrated respiratory alkalosis based on decreased PaCO_2_, metabolic alkalosis based on decreased A_TOT_, and metabolic acidosis based on decreased SIDa. We could diagnose metabolic alkalosis or acidosis with a normal anion gap after comparing delta A_TOT_ [(14.09 − measured A_TOT_) or (11.77 − 2.64 × Alb (g/dL))] and delta SIDa [(42.7 − measured SIDa) or (42.7 − (Na + K − Cl)]). We could also identify metabolic acidosis with an increased anion gap using SIG > 7.0 (SIG = 0.9463 × corrected anion gap—8.1956).

**Conclusions:**

Patients with nephrotic syndrome had primary respiratory alkalosis, decreased A_TOT_ due to hypoalbuminemia (power to metabolic alkalosis), and decreased levels of SIDa (power to metabolic acidosis). We could detect metabolic acidosis with an increased anion gap by calculating SIG. The Stewart model in combination with the Boston model facilitates the analysis of complex acid–base disturbances in nephrotic syndrome.

## Introduction

Several methods for analyzing acid–base disturbances have been proposed. One method is the traditional Boston model based on the CO_2_/HCO_3_ method [1, 2]. Another is the Copenhagen-Danish model based on base excess (BE) [3, 4]. A third method is the Stewart model based on the physicochemical method [5–7]. The Boston model makes it easy for beginners to understand and analyze acid–base disturbances. However, without the use of compensatory formulas, this model is limited in the setting of complex conditions such as respiratory abnormalities and two or more concurrent metabolic abnormalities. The BE model is useful only in metabolic acidosis. In contrast, the Stewart model is theoretically superior to the other models mentioned above in complex situations because it is based on physicochemical data. However, it is very difficult for physicians to understand the underlying concepts and make calculations using the relevant formulas in clinical practice. The Stewart model emphasizes the importance of albumin (Alb), which carries a negative charge in serum and influences acid–base balance; Alb is not taken into account by the traditional Boston model. There have been no reports about acid–base disturbances in nephrotic syndrome. In this study, we compared data derived using the Stewart model to data from the traditional Boston model in the context of acid–base disturbances in patients with nephrotic syndrome.

## Materials and methods

This study was approved by the Ethics Committee of Aichi Medical University (14-164).

### Patient characteristics (Table 1)

**Table 1:** Characteristics and laboratory data of the study patients

Patient	Age	Gender	Diagnosis		U-protein	TP	Albumin	BUN	Cr	eGFR	IgG	IgA	IgM
	(years)		Renal biopsy	Association	g/day	g/dL	g/dL	mg/dL	mg/dL	ml/min	mg/dL	mg/dL	mg/dL
1	47	F	Not done	SLE	2.1	4.5	1.3	9.4	0.41	126	447	597	75
2	37	F	MCNS		4.1	5.5	1.9	12.6	0.48	114	1677	264	125
3	55	M	Not done	Rectal cancer	5.2	5.2	2.1	11.6	0.58	111	1020	190	86
4	61	F	endo-GN		9.6	5.0	1.8	12.4	0.59	78	818	336	115
5	32	F	endo-GN		7.6	4.6	1.9	13.5	0.71	77	715	346	103
6	70	M	MN		7.0	5.7	2.9	12.0	0.77	76	712	264	92
7	71	F	MCNS		10.2	5.2	2.4	17.2	0.65	68	633	270	85
8	61	M	MCNS		14.3	4.8	1.0	21.9	0.92	65	1011	687	111
9	76	F	MCNS		10.7	3.9	0.9	34.8	0.73	58	746	154	36
10	65	M	MCNS		3.7	4.8	1.4	11.6	1.01	58	542	546	144
11	81	F	FSGS		7.0	5.5	2.8	25.5	0.75	56	541	165	45
12	78	F	MCNS		4.8	4.4	1.8	11.1	0.81	52	366	218	87
13	75	M	MCNS		10.7	3.6	1.1	33.5	1.10	51	422	244	33
14	89	F	Not done		4.2	4.7	2.4	16.4	0.81	50	687	203	58
15	70	M	MN		8.0	5.6	2.9	18.9	1.20	47	633	241	43
16	60	M	Not done	Thrombocytosis	11.0	4.5	1.9	18.7	1.46	40	866	224	40
17	60	M	FSGS		18.2	3.5	0.9	37.2	1.72	33	467	412	114
18	81	M	MN		5.7	4.2	1.8	25.4	1.61	33	604	224	74
19	87	F	Not done	MN suspected	6.5	4.6	1.8	24.2	1.20	33	734	529	21
20	71	F	Not done	MCNS suspected	12.3	4.4	1.4	78.0	1.38	30	362	314	123
21	86	M	Not done	MCNS suspected	3.9	4.1	0.8	27.9	1.76	29	1590	488	39
22	71	M	Not done	RA, bucillamine	7.5	5.1	2.1	45.8	1.97	27	883	60	104
23	54	M	Not done	DM	12.0	3.9	1.5	16.8	2.56	22	311	156	38
24	76	F	Not done		17.3	5.5	1.0	20.5	2.02	19	2755	414	42
25	76	M	Not done	AL amyloidosis	4.6	4.1	1.8	46.0	3.57	14	647	48	19
26	77	F	Crescentic GN		3.6	5.4	1.8	45.7	3.77	10	1290	285	42
27	82	M	Not done	DM	7.8	5.4	2.4	69.8	5.12	9	1022	192	58
28	74	F	Crescentic GN		4.8	5.5	2.0	70.9	4.13	9	1146	362	52
29	70	F	Not done	MPO-ANCA	3.9	5.3	1.9	69.3	5.64	6	2285	636	108
Median	71				7.0	4.8	1.8	21.9	1.2	47	715	264	74
25%	61				4.6	4.4	1.4	13.5	0.75	27	542	203	42
75%	77				10.7	5.4	2.1	37.2	1.97	65	1020	412	104

*MCNS* minimal change nephrotic syndrome, *endo-GN* endocapillary proliferative glomerulonephritis, *MN* membranous nephropathy, *FSGS* focal segmental glomerulosclerosis, *RA* rheumatoid arthritis, *DM* diabetes mellitus, *AL* amyloid light chain, *crescentic GN* crescentic glomerulonephritis, *MPO-ANCA* myeloperoxidase anti-neutrophil cytoplasmic antibody, *U-protein* urinary protein

We enrolled 29 patients with nephrotic syndrome and 6 patients without urinary protein or acid–base disturbances as a control group. All patients were Japanese. They were admitted to Aichi Medical University Hospital between August 2014 and August 2015. The diagnosis of nephrotic syndrome was based on massive proteinuria and hypoalbuminemia with or without edema. The study population included 14 men and 15 women, with a median age of 71 years [interquartile range (IQR), 61–77]. Sixteen patients had pathological diagnoses based on kidney biopsy: 7 patients with minimal change nephrotic syndrome (MCNS), 2 with focal segmental glomerulosclerosis (FSGS), 3 with membranous nephropathy (MN), 2 with endocapillary proliferative glomerulonephritis (endo-PGN), and 2 with crescentic glomerulonephritis (crescentic GN). The remaining 13 patients did not undergo kidney biopsy due to comorbidities or refusal to provide consent. Based on clinical and laboratory findings, these patients were suspected of having the following diagnoses: MCNS (*n* = 2), MN (*n* = 3; 1 with primary MN and 2 with MN secondary to malignancy and bucillamine), lupus nephritis (*n* = 1), essential thrombocytosis (*n* = 1), diabetes mellitus (*n* = 2), systemic amyloidosis (amyloid light chain type) (*n* = 1), anti-myeloperoxidase (MPO) antineutrophil cytoplasmic antibody (ANCA)-associated nephritis (*n* = 1), and no primary disease (*n* = 1). The median levels of urinary protein, serum total protein (TP), and serum Alb were 7.0 g/day (IQR, 4.6–10.7), 4.8 g/dL (4.4–5.4), and 1.8 g/dL (1.4–2.1), respectively. The median blood urea nitrogen (BUN) and creatinine (Cr) levels and estimated glomerular filtration rate (eGFR) were 21.9 mg/dL (13.5–37.2), 1.2 mg/dL (0.75–1.97), and 47.0 ml/min/1.72 m^2^ (27–65), respectively. The median immunoglobulin G (IgG), IgA, and IgM concentrations were 715 mg/dL (542–1020), 264 mg/dL (203–412), and 74 mg/dL (42–104), respectively (Table 1).

### Methods

Serum and urine samples were obtained from all patients with nephrotic syndrome before steroid and diuretic administration.

Laboratory examinations included TP (g/dL), Alb (g/dL), BUN (mg/dL), Cr (mg/dL), eGFR (mL/min/1.73 m^2^), Na (mEq/L), K (mEq/L), Cl (mEq/L), Ca (mg/dL), phosphate (P) (mg/dL), magnesium (Mg) (mg/dL), lactic acid (lactate) (mmol/L), IgG (mg/dL), IgA (mg/dL), IgM (mg/dL), plasma osmolarity (mOsm/L), arterial blood gas analysis, plasma renin activity (PRA) (ng/mL/h), and plasma aldosterone concentration (PAC) (pg/mL). Urinary examination was performed to evaluate daily urinary protein (g/day), urinary protein concentration (mg/dL), urine osmolarity (mOsm/L), Na (mEq/L), K (mEq/L), Cl (mEq/L), and Cr (mg/dL).

The anion gap (AG) in blood was calculated using the formula Na − (Cl + HCO_3_). The normal range was 12 ± 2 mEq/L. Corrected AG (cAG) was calculated using the formula [AG + 2.5 × (4.4 − measured albumin (g/dL))] according to the methods of Nguyen et al. [8] and Rastegar [9].

### CO_2_/HCO_3_ method (traditional Boston model)

Initially, we defined acidemia or alkalemia based on a pH of 7.40. Metabolic or respiratory disturbances, and acidosis or alkalosis, were determined using the CO_2_/HCO_3_ method. In respiratory alkalosis, we diagnosed acute or chronic respiratory alkalosis by comparing actual HCO_3_ to estimated HCO_3_ using Kellum’s compensatory formula [10]. Thus, estimated HCO_3_ = 0.2 × (actual PaCO_2_) + 17 in acute respiratory alkalosis and estimated HCO_3_ = 0.5 × (actual PaCO_2_) + 5 in chronic respiratory alkalosis. When there was a discrepancy between actual and estimated HCO_3_, we determined if additional metabolic acidosis or alkalosis was present.

### Physicochemical method (Stewart model)

We first evaluated several formulas reported by Nguyen et al. in 2009 [8], Rastegar in 2009 [9], Kishen et al. in 2014 [11], and Agrafiotis et al. in 2014 [12]. The Stewart model includes the following parameters: carbon dioxide (PaCO_2_); total concentration of non-volatile weak acids (A_TOT_), which is the sum of all buffer pairs (mostly weak acids) that move toward equilibrium with a dissociated anion [A^−^] according to its dissociation constant (e.g., albumin with its net negative charge under physiological conditions); and strong ion difference (SIG). The formulas for A_TOT_ reported by Nguyen et al. [8] and Kishen et al. [11] were incorrect. The formula should be A_TOT_ = Alb (g/dL) × 10 × (0.123 × pH − 0.631) + P (mg/dL) × 0.3229 × (0.309 × pH – 0.625) according to Rastegar [9], or A_TOT_ = Alb (g/dL) × 10 × (0.1204 × pH − 0.625) + P (mg/dL) × 0.3229 × (0.309 × pH − 0.469) according to Agrafiotis et al. [12]. Regarding the apparent strong ion difference (SIDa), the values for Ca^2+^, Mg^2+^, and lactate are negligible. The formula described for effective strong ion difference (SIDe) by Nguyen et al. and Kishen et al. (SIDe = [HCO_3_^−^] + A_TOT_) was incorrect because of errors in their formulas for A_TOT_. The units for A_TOT_, SIDa, SIDe, and SIG should be mEq/L, but not mmol/L as described by Kishen et al. [HCO_3_^−^] in SIDe is calculated as 2.46 × 10^−8^ × PCO_2_/10^(−pH)^ according to Kellum [13] or as 0.0301 × PCO_2_ × 10^(pH−6.1)^ according to Agrafiotis et al. [12]. Ultimately, we decided to use the formulas from Agrafiotis et al. [12]:

A_TOT_ (mEq/L) = [Alb (g/dL)] × 10 × (0.1204 × pH − 0.625) + [P (mg/dL)] × 0.3229 × (0.309 × pH − 0.469).

SIDa (mEq/L) = [Na^+^] + [K^+^] − [Cl^−^].

SIDe (mEq/L) = [HCO_3_^−^] + A_TOT_ = [0.0301 × PCO_2_ × 10^(pH−6.1)^] + A_TOT_.

SIG (mEq/L) = SIDa − SIDe.

### Relationships between albumin and A_TOT_ and between cAG and SIG

We created scatter plots with albumin or cAG on the *x*-axis and A_TOT_ or SIG on the *y*-axis. We also derived correlation coefficients using Excel (Microsoft Corporation, Redmond, WA).

### Relationship between eGFR and SIG or (SIG − lactate)

We created scatter plots with eGFR on the *x*-axis, and SIG or (SIG − lactate) on the *y*-axis. (SIG − lactate) represents unknown non-volatile acids other than lactate. We also derived correlation coefficients using Excel (Microsoft Corporation).

### Relationship between IgG and A_TOT_, SIDa, SIDe, and SIG

We created scatter plots with IgG on the *x*-axis and A_TOT_, SIDa, SIDe, or SIG on the *y*-axis. We also derived correlation coefficients using Excel (Microsoft Corporation).

## Results

### Serum electrolytes (Table 2)

**Table 2:** Summary of laboratory data in the nephrotic syndrome and control groups

	Unit	Nephrotic syndrome group		Control group	
		Median	25–75%	Median	25–75%
Albumin	g/dL	1.8	1.4–2.1	4.7	4.5–4.8
Na	mEq/L	140	137–142	142	140–145
K	mEq/L	4.1	3.7–4.5	4.7	4.5–4.8
Cl	mEq/L	107	104–110	105	103–105
Na-Cl	mEq/L	33	31–34	37	37–39
Na + K-Cl	mEq/L	36.4	35.0–38.0	42.7	41.9–43.3
Ca	mg/dL	7.7	7.1–8.1	9.6	9.4–9.8
cCa	mg/dL	9.8	9.6–10.0	9.6	9.4–9.8
P	mg/dL	3.8	3.3–4.2	3.4	3.0–3.8
Mg	mg/dL	1.9	1.7–2.0	2.1	2.0–2.2
Lactate	mmol/L	1.3	1.0–1.9	0.9	0.8–1.0
pH		7.43	7.42–7.45	7.40	7.39–7.41
PaCO_2_	Torr	34.9	30.8–40.0	40.4	39.4–41.1
PaO_2_	Torr	84.2	70.3–98.8	89.5	87.5–90.1
HCO_3_	mEq/L	23.7	21.1–26.0	25.1	24.2–25.3
A-aDO_2_	Torr	33.9	28.4–53.6	10.8	9.6–11.4
AG	mEq/L	8.6	7.0–10.8	13.5	13.0–14.5
cAG	mEq/L	15.8	13.6–17.4	13.1	12.5–13.5
A_TOT_	mEq/L	7.13	6.00–7.83	14.48	13.73–14.57
SIDa	mEq/L	36.4	35.0–38.0	42.7	41.9–43.3
SIDe	mEq/L	29.9	27.1–32.9	38.7	38.5–39.3
SIG	mEq/L	6.6	4.4–8.2	3.9	3.4–5.1
Posm	mOsm/L	287	284–294	nd	nd
Uosm	mOsm/L	466	296–592	nd	nd
U-Na	mEq/L	60	37–111	nd	nd
U-K	mEq/L	31.1	20.2–51.5	nd	nd
U-Cl	mEq/L	61	31–101	nd	nd
Urinary AG	mEq/L	38.9	29.0–55.0	nd	nd

*cCa* corrected Ca, *AG* anion gap, *cAG* corrected anion gap, *A*_*TOT*_ nonvolatile weak acids, *SIDa* apparent strong ion difference, *SIGc* corrected strong ion gap, *Posm* plasma osmolarity, *Uosm* urinary osmolarity, *U-Na* urinary Na, *U-K* urinary K, *U-Cl* urinary Cl

The median serum concentrations of Na, K, and Cl were 140 mEq/L (IQR, 137–142), 4.1 (3.7–4.5), and 107 mEq/L (104–110), respectively. The concentration of serum K was slightly lower than in the control group [4.7 mEq/L (4.5–4.8)]. The median actual Ca concentration was significantly lower at 7.7 mg/dL (7.1–8.1); however, the median Ca concentration corrected for serum albumin was within the normal range at 9.8 mg/dL (9.6–10.0). The median P and Mg levels were 3.8 (3.3–4.2) and 1.9 mg/dL (1.7–2.0), respectively. The median lactate level was 1.3 mmol/L (1.0–1.9), which slightly higher than in the control group [0.9 mmol/L (0.8–1.0)]. The median IgG, IgA, and IgM concentrations were 715 (542–1020), 264 (203–412), and 74 mg/dL (42–104), respectively.

### Traditional model (Boston model)

The median pH was slightly alkalotic at 7.43 (IQR, 7.42–7.45) (Tables 2, 3); 25 of 29 patients had a pH of more than 7.40, while 4 had a pH of less than 7.40. The median PaCO_2_ was 34.9 Torr (30.8–40.0). Twenty-two patients had PaCO_2_ levels of less than 40 Torr, while 7 had levels between 40 and 44.7 Torr. The median A-aDO_2_ was 33.9 Torr [28.4–53.6]. Given that the normal limit of A-aDO_2_ is less than 20 Torr, 26 of 29 patients (90%) in the high age group had impaired blood gas diffusion. The median PaO_2_ was 84.2 Torr [70.3–99.8]. These results suggest that hyperventilation occurs in most nephrotic patients. Since we routinely exclude pulmonary embolism and infection using laboratory data and chest computed tomography, hypoalbuminemia due to nephrotic syndrome seems to induce pulmonary interstitial edema, which in turn increases A-aDO_2_. Hyperventilation then occurs in order to maintain PaO_2_ levels. Primary respiratory alkalosis is essential in patients with nephrotic syndrome. The median HCO_3_ concentration was 23.7 mEq/L [21.1–26.0]. In cases of primary respiratory alkalosis, HCO_3_ concentrations should be decreased as a result of compensatory mechanisms. Actual HCO_3_ concentrations that are higher than expected suggest the coexistence of metabolic alkalosis. In nephrotic syndrome, the analysis of acid–base disturbances using the Boston method is limited because it requires the use of Kellum’s compensatory formula for respiratory alkalosis [10] or the concept of cAG.

**Table 3:** Blood gas analysis

Patient	pH	PaCO_2_	PaO_2_	HCO_3_	A-aDO_2_	AG	cAG	Lactate
		Torr	Torr	mEq/L	Torr	mEq/L	mEq/L	mmol/L
1	7.43	34.0	70.3	22.1	46.8	8.9	16.7	1.3
2	7.48	25.7	110.6	18.9	16.9	11.1	17.4	0.6
3	7.46	34.1	128.3	23.7	79.9	8.3	14.1	1.2
4	7.44	38.7	82.8	26.0	28.4	6.0	12.5	2.1
5	7.45	36.4	106.6	24.8	7.5	5.2	11.5	1.0
6	7.45	35.0	47.3	24.0	68.6	7.0	10.8	1.6
7	7.43	37.4	83.3	24.4	29.6	8.6	13.6	0.9
8	7.44	35.1	98.3	23.5	17.4	7.5	16.0	3.3
9	7.55	27.9	66.6	24.2	58.1	10.8	19.6	2.4
10	7.43	43.2	84.2	28.2	21.4	7.8	15.3	1.8
11	7.34	34.9	85.5	21.2	30.5	11.8	15.8	4.2
12	7.43	41.3	75.7	28.0	32.3	6.0	12.5	0.9
13	7.42	40.8	67.2	25.9	41.4	5.1	13.4	1.5
14	7.56	29.4	92.2	26.1	30.7	9.9	14.9	1.3
15	7.42	42.1	83.6	26.8	23.4	9.2	13.0	1.0
16	7.36	40.0	56.0	22.4	53.6	9.6	15.9	2.6
17	7.47	33.1	80.4	23.7	37.8	7.3	16.1	1.4
18	7.45	36.9	84.8	24.9	28.7	7.1	13.6	0.9
19	7.43	43.9	99.5	28.6	5.2	7.4	13.9	3.8
20	7.42	41.3	59.3	26.1	48.7	9.9	17.4	1.5
21	7.49	28.4	114.4	21.1	100.9	6.9	15.9	1.2
22	7.42	32.0	94.1	20.6	25.5	13.4	19.2	1.7
23	7.43	44.6	62.7	29.4	41.2	5.6	12.9	1.0
24	7.45	30.8	52.4	20.9	68.7	6.1	14.6	1.2
25	7.45	31.9	125.4	21.9	55.1	10.1	16.6	1.2
26	7.48	26.5	77.9	19.3	48.6	13.7	20.2	1.0
27	7.29	31.5	87.9	14.9	32.3	14.1	19.1	0.9
28	7.32	28.7	111.5	14.1	73.0	16.9	22.9	1.9
29	7.40	21.5	98.8	13.1	33.9	16.9	23.2	1.9
Median	7.43	34.9	84.2	23.7	33.9	8.6	15.8	1.3
25%	7.42	30.8	70.3	21.1	28.4	7.0	13.6	1.0
75%	7.45	40.0	98.8	26.0	53.6	10.8	17.4	1.9

*AG* anion gap, *cAG* corrected anion gap

### Serum AG (Tables 2, 3)

The median AG was 8.6 mEq/L (IQR, 7.0–10.8), which was significantly lower than the normal range of 12 ± 2 mEq/L. The median cAG, which adjusts for serum albumin using the formula cAG = AG + 2.5 × (4.4 − measured albumin (g/dL)), was 15.8 mEq/L (13.6–17.4). Twelve patients had cAG values within the normal range of 11.0–15.1 mEq/L, 16 patients had increased levels greater than 15.1 mEq/L, and only one patient had a slightly decreased level of 10.8 mEq/L (Table 3).

### PRA and PAC (Table 4)

**Table 4:** Plasma and urine osmolarity, urinary electrolytes, plasma renin activity, and plasma aldosterone concentration

Patient	Posm	Uosm	U-Na	U-K	U-Cl	Urinary AG	Renin	P-Ald
	mOsm/L	mOsm/L	mEq/L	mEq/L	mEq/L		ng/mL/h	pg/mL
1	276	810	20	54.8	46	28.8	19.0	618.0
2	283	722	80	52.0	96	36.0	0.6	10.0
3	284	499	152	19.0	141	30.0	1.1	39.1
4	287	528	117	34.6	90	61.6	0.1	10.0
5	282	592	127	46.2	141	32.2	0.2	40.1
6	281	290	53	22.0	49	26.0	13.0	191.0
7	293	602	155	28.4	133	50.4	0.4	70.1
8	290	818	42	68.4	108	2.4	16.0	309.0
9	298	749	81	39.2	81	39.2	18.0	77.4
10	285	417	39	20.1	31	28.1	1.5	67.8
11	306	432	113	17.0	101	29.0	0.8	101.0
12	285	244	114	100.0	159	55.0	0.6	10.0
13	294	813	36	61.9	39	58.9	2.6	45.0
14	286	466	105	32.3	87	50.3	0.7	27.9
15	294	475	85	31.1	72	44.1	1.6	50.4
16	282	328	138	26.9	126	38.9	0.5	19.2
17	293	560	56	30.1	21	65.1	0.4	10.0
18	286	484	37	51.5	22	66.5	0.9	33.6
19	301	392	111	32.3	86	57.3	0.8	10.0
20	332	796	18	72.3	12	78.3	0.7	16.4
21	281	453	26	42.9	25	43.9	0.5	32.1
22	287	474	62	24.5	46	40.5	13.0	85.6
23	291	296	109	14.7	110	13.7	0.4	10.0
24	267	264	33	27.4	31	29.4	0.4	26.7
25	297	275	33	16.6	16	33.6	2.9	20.6
26	285	248	48	20.2	40	28.2	0.2	53.1
27	310	431	35	57.1	25	67.1	1.2	50.8
28	302	274	41	19.1	24	36.1	0.3	59.1
29	286	196	60	15.8	61	14.8	0.1	72.4
Median	287	466	60	31.1	61	38.9	0.7	40.1
25%	284	296	37	20.2	31	29.0	0.4	19.2
75%	294	592	111	51.5	101	55.0	1.6	70.1

*P-Ald* plasma aldosterone concentration, *Posm* plasma osmolarity, *Uosm* urinary osmolarity, *U-Na* urinary Na, *U-K* urinary K, *U-Cl* urinary Cl

PRA values were within the normal range (0.3–2.9 ng/mL/h) in 19 patients, below normal in 5 patients, and above normal in 5 patients. PAC was within the normal range (30–160 pg/mL) in 15 patients. Hypoaldosteronism was observed in 11 patients, and 3 patients had hyperreninemic hyperaldosteronism. These results demonstrated that hypoaldosteronism and normoaldosteronism are the predominant conditions in nephrotic syndrome.

### Stewart model (Table 5)

**Table 5:** Data from the Stewart model

Patient	pH	PaCO2	A_TOT_	Delta A_TOT_ (to alkalosis)	SIDa	Delta SIDa (to acidosis)	Normal AG metabolic	SIDe	SIG	Increased AG acidosis	Lactate	SIG-lactate
		Torr	14.09		42.7			38.9	1.4–7.0		mmol/L	mEq/L
1	7.43	34.00	6.16	7.93	34.8	7.9	Neutral	28.1	6.7		1.3	5.4
2	7.48	25.70	7.69	6.40	34.1	8.6	Acidosis	26.4	7.7	AG+	0.6	7.0
3	7.46	34.10	7.69	6.40	35.8	6.9	Acidosis	31.2	4.6		1.2	3.5
4	7.44	38.70	7.13	6.96	36.5	6.2	Alkalosis	32.8	3.7		2.1	1.6
5	7.45	36.40	7.47	6.62	34.4	8.3	Acidosis	31.9	2.5		1.0	1.5
6	7.45	35.00	9.49	4.60	35.2	7.5	Acidosis	33.2	2.0		1.6	0.4
7	7.43	37.40	8.60	5.49	37.1	5.6	Acidosis	32.8	4.3		0.9	3.5
8	7.44	35.10	5.02	9.07	35.1	7.6	Alkalosis	28.2	6.9		3.3	3.6
9	7.55	27.90	4.91	9.18	38.0	4.7	Alkalosis	28.7	9.3	AG+	2.4	6.8
10	7.43	43.20	5.02	9.07	40.6	2.1	Alkalosis	33.0	7.6	AG+	1.8	5.8
11	7.34	34.90	8.93	5.16	37.3	5.4	Acidosis	27.1	10.2	AG+	4.2	6.0
12	7.43	41.30	6.67	7.42	37.9	4.8	Alkalosis	33.1	4.8		0.9	3.9
13	7.42	40.80	5.25	8.84	35.3	7.4	Alkalosis	30.9	4.4		1.5	2.9
14	7.56	29.40	8.91	5.18	39.7	3.0	Alkalosis	34.7	5.0		1.3	3.7
15	7.42	42.10	10.44	3.65	40.3	2.4	Alkalosis	37.0	3.3		1.0	2.3
16	7.36	40.00	7.83	6.26	36.5	6.2	Neutral	29.9	6.6		2.6	4.0
17	7.47	33.10	3.89	10.20	34.5	8.2	Alkalosis	27.2	7.3	AG+	1.4	6.0
18	7.45	36.90	6.84	7.25	36.4	6.3	Alkalosis	31.5	4.9		0.9	4.0
19	7.43	43.90	7.04	7.05	39.5	3.2	alkalosis	35.4	4.1		3.8	0.4
20	7.42	41.30	7.05	7.04	41.1	1.6	Alkalosis	32.9	8.2	AG+	1.5	6.7
21	7.49	28.40	4.24	9.85	31.7	11.0	Acidosis	25.3	6.4		1.2	5.2
22	7.42	32.00	7.77	6.32	38.9	3.8	Alkalosis	28.1	10.8	AG+	1.7	9.1
23	7.43	44.60	6.00	8.09	38.5	4.2	Alkalosis	35.0	3.5		1.0	2.5
24	7.45	30.80	5.20	8.89	30.7	12.0	Acidosis	25.8	4.9		1.2	3.6
25	7.45	31.90	7.27	6.82	36.5	6.2	Alkalosis	29.0	7.5	AG+	1.2	6.4
26	7.48	26.50	6.69	7.40	36.0	6.7	Alkalosis	25.9	10.1	AG+	1.0	9.1
27	7.29	31.50	8.89	5.20	35.0	7.7	Acidosis	23.6	11.4	AG+	0.9	10.4
28	7.32	28.70	8.53	5.56	36.4	6.3	Acidosis	22.8	13.6	AG+	1.9	11.7
29	7.40	21.50	7.64	6.45	33.9	8.8	Acidosis	20.6	13.3	AG+	1.9	11.4
Median	7.43	34.90	7.13	6.96	36.4	6.3		29.9	6.6		1.3	
25%	7.42	30.80	6.00	6.26	35.0	4.7		27.1	4.4		1.0	
75%	7.45	40.00	7.83	8.09	38.0	7.7		32.9	8.2		1.9	

Control group mean values: A_TOT_, 14.09; SIDa, 42.7; SIG, 4.2; lactate, 0.9

In the control group, the median A_TOT_, SIDa, SIDe, and SIG values were 14.48 (IQR, 13.73–14.57), 42.7 (41.9–43.3), 38.7 (38.5–39.3), and 3.9 mEq/L (3.4–5.1), respectively. The mean ± SD for A_TOT_, SIDa, SIDe, and SIG were 14.09 ± 0.90 (normal range: 12.29–15.90), 42.7 ± 1.4 (normal range: 39.9–45.5), 38.5 ± 1.2 (normal range: 36.1–41.0), and 4.2 ± 1.4 (normal range: 1.4–7.0), respectively.

#### PaCO_2_ (Torr)

**Fig. 1:**
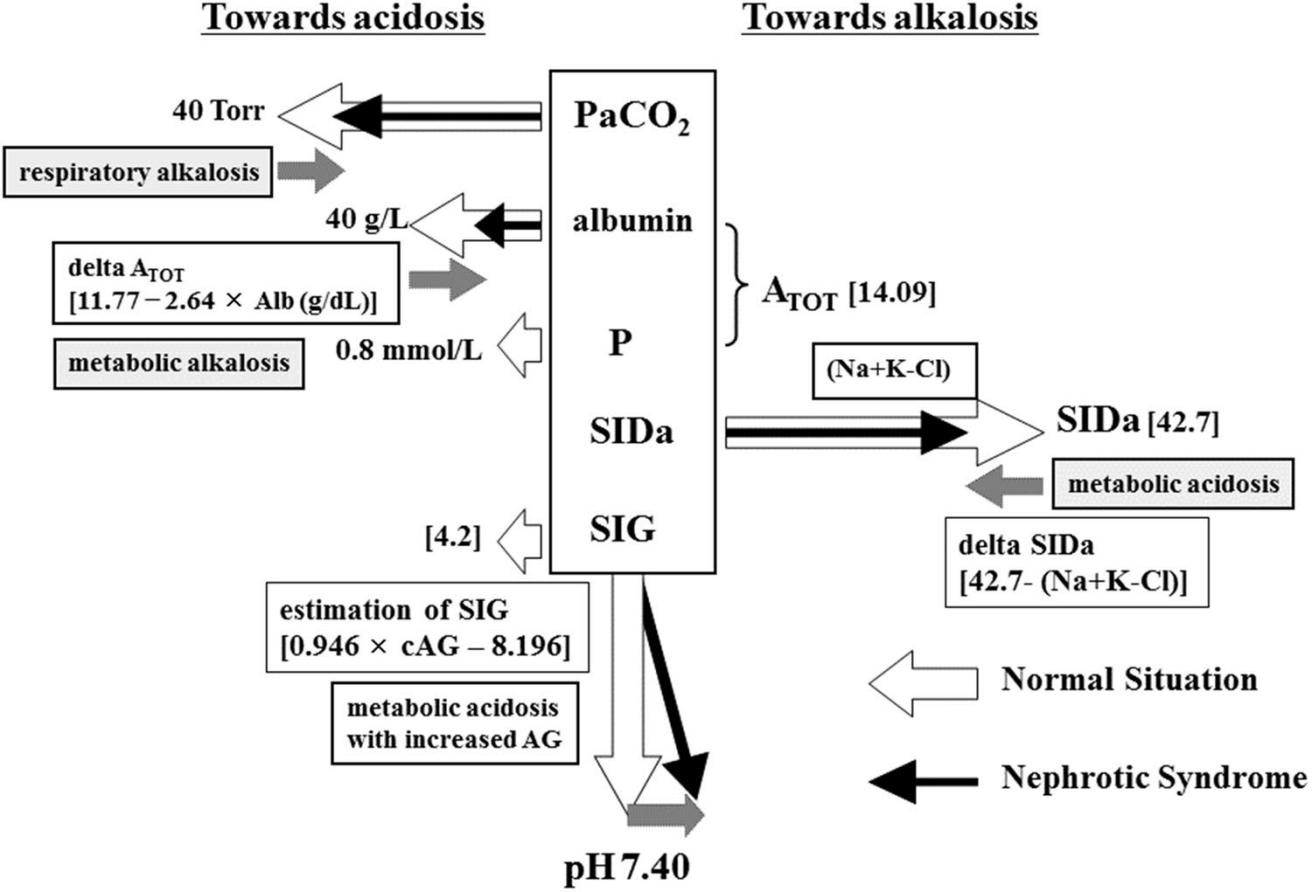
Determination of pH in the Stewart model. In the Stewart model, PaCO_2_, A_TOT_, and SIGc have powers toward acidosis. On the other hand, SIDa has a power toward alkalosis. In healthy individuals, pH should be 7.40. In nephrotic syndrome, patients are alkalotic due to decreased levels of PaCO_2_, decreased A_TOT_ due to hypoalbuminemia, and decreased SIDa resulting from hyperchloremia due to hyporeninemic hypoaldosteronism

The median PaCO_2_ level was 34.9 Torr (IQR, 30.8–40.0). PaCO_2_ was less than 40 Torr in 21 of 29 patients (76%). Low PaCO_2_ levels, such as those observed here, contribute to the development of alkalosis (Fig. 1).

#### A_TOT_ (mEq/L)

A_TOT_ represents the total concentration of non-volatile weak acids. It depends on albumin and phosphate levels. The median A_TOT_ was 7.13 (mEq/L) (IQR, 6.00–7.83), because hypoalbuminemia secondary to nephrotic syndrome directly interferes with A_TOT_ values. The median delta A_TOT_, calculated by [14.09 (mean value of control group) − A_TOT_], was 6.96 (6.26–8.09). This lower value of A_TOT_ contributed to metabolic alkalosis (Fig. 1).

#### SIDa (mEq/L)

SIDa reflects the likelihood of alkalosis. The median SIDa value, calculated by [Na + K − Cl], was 36.4 (mEq/L) (IQR, 35.0–38.0), which was lower than the mean value in the control group (42.7 mEq/L). The median delta SIDa, calculated by [42.7 (mean value of control group) − SIDa], was 6.3 (mEq/L) (4.7–7.7). Lower SIDa among nephrotic patients suggests a power to metabolic acidosis (Fig. 1). In individual patients, delta A_TOT_ (alkalosis) greater than delta SIDa (acidosis) results in metabolic alkalosis. Inversely, when delta SIDa is greater than delta A_TOT_, the patient has metabolic acidosis with a normal anion gap.

#### SIDe (mEq/L)

The median SIDe was 29.9 mEq/L (IQR, 27.1–32.9), which was lower than the mean value in the control group (38.5 mEq/L).

#### SIG (mEq/L)

SIG reflects the accumulation of various non-volatile acids, including lactate or uremic substances. Increased AG suggests metabolic acidosis with an increased anion gap. The median SIG was 6.6 mEq/L (IQR, 4.4–8.2), which was higher than 3.9 mEq/L (3.4–5.1) in the control group. The mean ± SD in the control group was 4.2 ± 1.4 mEq/L (normal range: 1.4–7.0).

#### Lactate (mmol/L = mEq/L) and SIG (mEq/L)

The median lactate was 1.3 mmol/L (IQR, 1.0–1.9), higher than 0.9 mmol/L (0.8–1.0) in the control group. The mean was 0.9 mmol/L (normal range: 0.8–1.0). Lactate levels greater than 1.0 mmol/L were observed in 20 of 29 patients (69%), and 12 out of 29 patients had metabolic acidosis with an increased anion gap. (SIG − lactate) represents the accumulation of non-volatile acids except for lactate. Seven of 12 patients had mainly lactate accumulation. Three had accumulation of lactate and non-volatile acids such as uremic toxins. Two of 12 patients had accumulation of non-volatile acids except for lactate.

### Relationship between serum albumin and A_TOT_

**Fig. 2:**
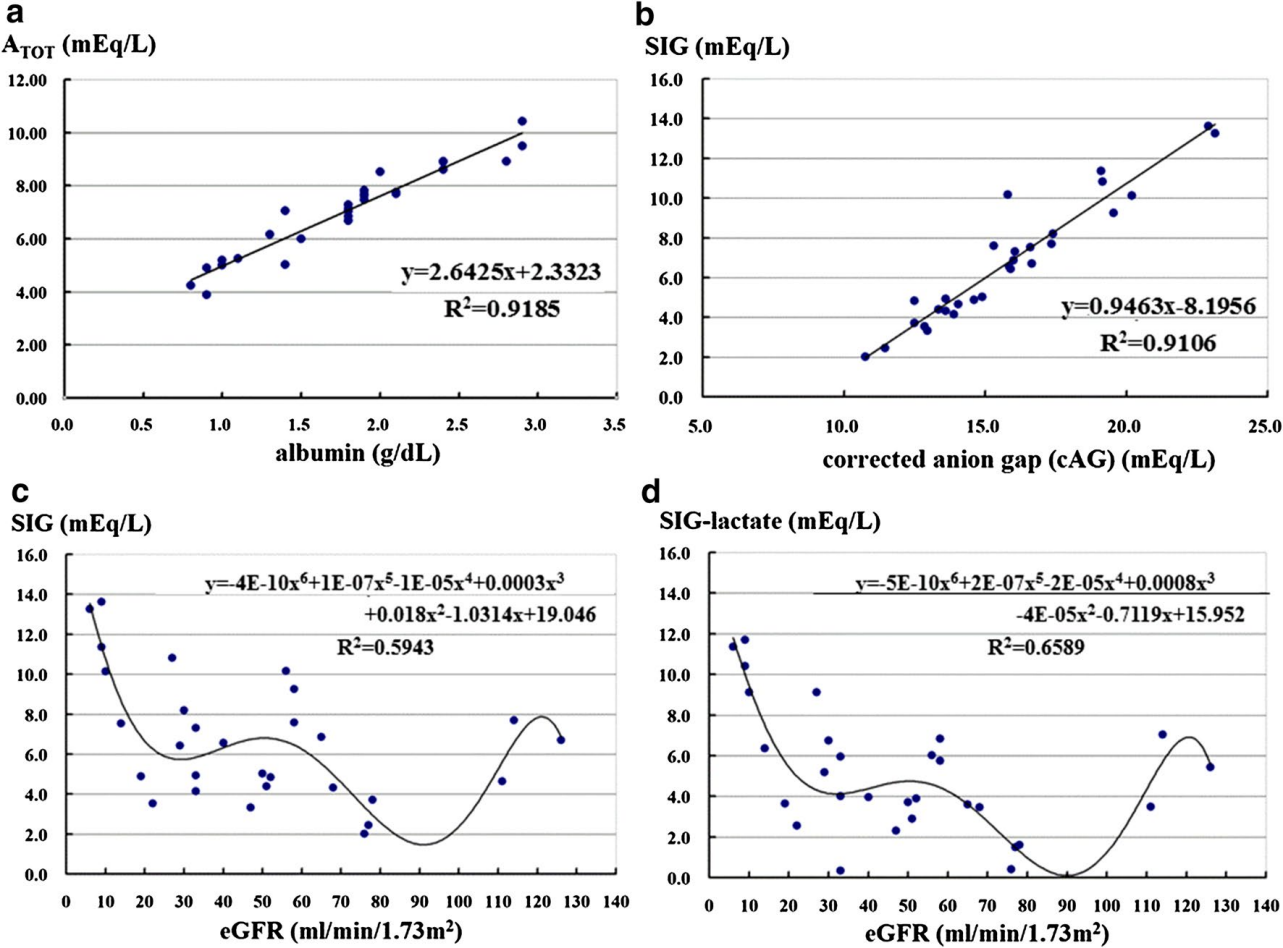
Relationship between albumin, A_TOT_, eGFR, and SIG. **a** Relationship between albumin and A_TOT_: A_TOT_ = 2.6425 × Alb + 2.3323 (*R* ^2^ = 0.91851). Delta A_TOT_ is calculated as 11.77 − 2.64 × Alb (g/dL). This number indicates the power to alkalosis. **b** Relationship between cAG and SIG: SIG = 0.9463 × cAG − 8.1956 (R^2^ = 0.91057). **c** Relationship between eGFR and SIG: SIG = − 4E-10(eGFR)^6^ + 1E−07(eGFR)^5^ − 1E−05(eGFR)^4^ + 0.0003(eGFR)^3^ + 0.018(eGFR)^2^ − 1.0314(eGFR) + 19.046 (R^2^ = 0.5943). **d** Relationship between eGFR and SIG − lactate: (SIG-lactate) = − 5E−10 (eGFR)^6^ + 2E-07 (eGFR)^5^ − 2E−05 (eGFR)^4^ + 0.0008 (eGFR)^3^ − 4E−05 (eGFR)^2^ − 0.7119 (eGFR) + 15.952 (*R* ^2^ = 0.6589) (**c**,** d**). Six out of 9 patients with eGFR less than 29 mL/min had metabolic acidosis with an increased anion gap. Five out of 6 patients had accumulation of lactate and unknown non-volatile acids (i.e., uremic toxins). One patient had increased levels of lactate alone. On the other hand, increased lactate levels were observed in patients with eGFR greater than 30 mL/min, who have metabolic acidosis and an increased anion gap

There was a strong significant relationship between serum albumin and A_TOT_: A_TOT_ = 2.6425 × Alb + 2.3323 (*R* ^2^ = 0.91851) (Fig. 2a). We can easily calculate and estimate A_TOT_ using serum albumin alone. When the serum albumin level is 4.4 g/dL, A_TOT_ will be 14.0, a normal value (14.09). If the albumin level is 1.5 g/dL, A_TOT_ will be 6.30. In addition, delta A_TOT_, which represents the power to metabolic alkalosis, is calculated using the formula 11.77 − 2.64 × Alb (g/dL) (Fig. 1).

### Relationship between cAG and SIG

We estimated SIG using the formula SIG = 0.9463 × cAG − 8.1956 (*R* ^2^ = 0.91057) (Fig. 2b). After calculating delta cAG [cAG − 12], the formula can be rewritten as SIG = 0.9463 × delta cAG + 3.1605 (*R*² = 0.91057).

### Relationship between eGFR and SIG or (SIG − lactate)

The relationship between eGFR and SIG is as follows: SIG = − 4E−10(eGFR)^6^ + 1E−07(eGFR)^5^ − 1E−05(eGFR)^4^ + 0.0003(eGFR)^3^ + 0.018(eGFR)^2^ − 1.0314(eGFR) + 19.046 (*R* ^2^ = 0.5943). The relationship between eGFR and (SIG − lactate) is as follows: (SIG − lactate) = − 5E−10(eGFR)^6^ + 2E−07(eGFR)^5^ − 2E−05(eGFR)^4^ + 0.0008(eGFR)^3^ − 4E−05(eGFR)^2^ − 0.7119(eGFR) + 15.952 (*R* ^2^ = 0.6589) (Fig. 2c, d). Six of 9 patients with eGFR less than 29 mL/min/1.72 m^2^ had metabolic acidosis with an increased anion gap. Five out of 6 patients had accumulation of lactate and unknown non-volatile acids (i.e., uremic toxins). One patient had increased lactate levels alone. On the other hand, patients with eGFR greater than 30 mL/min/1.72 m^2^ who had metabolic acidosis with an increased anion gap had increased lactate levels.

### Relationship between IgG and A_TOT_, SIDa, SIDe, and SIG

Levels of IgG, a positively charged protein, might influence acid–base balance. The relationship between IgG and A_TOT_, SIDa, SIDe, and SIG can be expressed as A_TOT_ = −0.0002 × (IgG) + 7.2633 (*R*² = 0.0073), SIDa = − 0.0029 × (IgG) + 39.076 (*R*² = 0.4337), SIDe = − 0.0046 × (IgG) + 33.834 (*R*² = 0.4105), and SIG = 0.0017 × (IgG) + 5.242 (*R*² = 0.0943), respectively. In other words, IgG levels have a negligible influence in the Stewart model.

## Discussion

This study revealed 4 specific findings about acid–base disturbances in nephrotic syndrome. First, primary respiratory alkalosis occurs due to respiratory dysfunction, with elevated A-aDO2 caused by pulmonary interstitial edema due to hypoalbuminemia. Second, the comparison between delta A_TOT_ and delta SID helps to determine whether metabolic alkalosis or metabolic acidosis with a normal anion gap is present. Third, we can estimate A_TOT_ using serum albumin, SIDa as Na + K − Cl, and SIG using cAG. Fourth, 90% of patients had hyporeninemic or normoreninemic hypoaldosteronism, not hyperreninemic hyperaldosteronism. The above findings suggest that hyperchloremic metabolic acidosis in nephrotic syndrome is due to hyporeninemic hypoaldosteronism, with a shift of K into the intracellular space due to alkalemia.

The Boston model is easy for beginners to understand and use to analyze acid–base disturbances. However, without the use of compensatory formulas described by Kellum [10], this model is limited in the setting of complex conditions such as respiratory abnormalities and 2 or more concurrent metabolic abnormalities. It is very hard not only for beginners, but also for nephrologists to understand and calculate the effects of compensatory mechanisms. On the other hand, the Stewart model is theoretically superior to the Boston model in complex situations because it is based on physicochemical data. Changes after treatment such as supplementation of solution or albumin can be evaluated and be estimated using the Stewart model. The present study revealed that the formula from Agrafiotis et al. [12] is the most reliable; several manuscripts including those by Nguyen et al. [8], Restegar [9], and Kishen et al. [11], contained incorrect formulas. A lack of consensus on formulas for the Stewart model limits its use. Masevicius and Dubin [14] claimed that the Stewart approach is cumbersome, requires more determinations and calculations, and is more time-consuming and expensive. They only need to continue with the proper use of the Boston model. However, this study suggested that a combination of the Boston and Stewart models would be important to consider in complicated cases such as mixed metabolic disorders and hypoalbuminemia.

In the Steward model, the first step in the approach to analyzing acid–base disturbances in nephrotic syndrome is calculating delta A_TOT_ and delta SIDa. The second step is comparing delta A_TOT_ and delta SIDa values. When delta A_TOT_ (14.1 − measured A_TOT_) is greater than delta SIDa (49.2 − measured SIDa), we can predict the presence of metabolic alkalosis. Inversely, when delta A_TOT_ is smaller than delta SIDa, metabolic acidosis with a normal anion gap is likely to be present. Thus, we can easily distinguish between metabolic alkalosis and metabolic acidosis using delta A_TOT_ and delta SIDa values. This study also demonstrated a strong relationship between A_TOT_ and serum albumin and between SIG and cAG. We can estimate A_TOT_ and delta A_TOT_ using the following formulas: A_TOT_ = 2.6425 × Alb + 2.3323 (*R* ^2^ = 0.91851) and delta A_TOT_ = 11.77 − 2.64 × Alb (g/dL) (power to alkalosis). Since SIDa is calculated as (Na + K − Cl), the formula for delta SIDa is (49.2 − measured SIDa), which indicates the power to acidosis. In addition, we can identify another metabolic acidosis with an increased anion gap using the formula SIG = 0.9463 × cAG − 8.1956 (*R* ^2^ = 0.91057). (more than 7.0). The reliability of the estimated formula is 89.7% (26/29) in metabolic alkalosis or acidosis with a normal anion gap and 86.2% (25/29) in metabolic acidosis with an increased anion gap (data not shown). Our formulas will help [who?] understand mixed acid–base disturbances in nephrotic syndrome and renal failure.

The present study revealed that patients with eGFR less than 29 mL/min/1.72 m^2^ mainly have accumulation of lactate and uremic toxins. On the other hand, patients with eGFR greater than 30 mL/min/1.72 m^2^ have metabolic acidosis with an increased anion gap as a result of increased lactate levels alone. Systemic edema due to nephrotic syndrome from MCNS or FSGS may influence the production of lactate.

Regarding the mechanism of edema in nephrotic syndrome, the underfill and overfill hypotheses have been proposed [15]. According to the underfill hypothesis, the renin–angiotensin–aldosterone system is activated by decreased intravascular blood volume, which in turn induces sodium retention and causes edema. However, the present study demonstrated that only 10% of patients have hyperreninemic hyperaldosteronism, while 90% had hypoaldosteronism or normoaldosteronism. These data support the overfill hypothesis [16, 17]. Fluid management should involve administration of albumin and loop diuretics. Anti-aldosterone drugs such as spironolactone and eplerenone should be avoided. Recently, acetazolamide and hydrochlorothiazide followed by furosemide have been reported to be more effective for the treatment of refractory nephrotic edema [18]. However, the influence of these treatments on acid–base disturbances in nephrotic syndrome should be further studied because acetazolamide induces metabolic acidosis by increasing urinary excretion of Na^+^ and HCO_3_^−^.

## Conclusion

We showed that patients with nephrotic syndrome have primary respiratory alkalosis, decreased A_TOT_ due to hypoalbuminemia (power to metabolic alkalosis), decreased SIDa (power to metabolic acidosis), and increased SIG, suggesting that they accumulate non-volatile acids such as lactate, uremic toxins, or other acids. Using both the Stewart and Boston methods facilitates the analysis of complex acid–base disturbances in primary and secondary nephrotic syndrome.
